# Adverse bioenergetic effects of N-acyl amino acids in human adipocytes overshadow beneficial mitochondrial uncoupling

**DOI:** 10.1016/j.redox.2023.102874

**Published:** 2023-09-02

**Authors:** Marie Herrnhold, Isabel Hamp, Oliver Plettenburg, Martin Jastroch, Michaela Keuper

**Affiliations:** a Department of Molecular Biosciences, The Wenner-Gren Institute, The Arrhenius Laboratories F3, Stockholm University, SE-106 91, Stockholm, Sweden; b Institute of Medicinal Chemistry, Helmholtz Zentrum München, German Research Center for Environmental Health (GmbH), Neuherberg, Germany; c Institute of Organic Chemistry, Leibniz Universität Hannover, Hannover, Germany

**Keywords:** Obesity, Metabolism, Mitochondria, UCP1, Adipocytes, Uncoupling

## Abstract

**Objective:**

Enhancing energy turnover via uncoupled mitochondrial respiration in adipose tissue has great potential to improve human obesity and other metabolic complications. However, the amount of human brown adipose tissue and its uncoupling protein 1 (UCP1) is low in obese patients. Recently, a class of endogenous molecules, N-acyl amino acids (NAAs), was identified as mitochondrial uncouplers in murine adipocytes, presumably acting via the adenine nucleotide translocator (ANT). Given the translational potential, we investigated the bioenergetic effects of NAAs in human adipocytes, characterizing beneficial and adverse effects, dose ranges, amino acid derivatives and underlying mechanisms.

**Method:**

NAAs with neutral (phenylalanine, leucine, isoleucine) and polar (lysine) residues were synthetized and assessed in intact and permeabilized human adipocytes using plate-based respirometry. The Seahorse technology was applied to measure bioenergetic parameters, dose-dependency, interference with UCP1 and adenine nucleotide translocase (ANT) activity, as well as differences to the established chemical uncouplers niclosamide ethanolamine (NEN) and 2,4-dinitrophenol (DNP).

**Result:**

NAAs with neutral amino acid residues potently induce uncoupled respiration in human adipocytes in a dose-dependent manner, even in the presence of the UCP1-inhibitor guanosine diphosphate (GDP) and the ANT-inhibitor carboxyatractylate (CAT). However, neutral NAAs significantly reduce maximal oxidation rates, mitochondrial ATP-production, coupling efficiency and reduce adipocyte viability at concentrations above 25 μM. The *in vitro* therapeutic index (using induced proton leak and viability as determinants) of NAAs is lower than that of NEN and DNP.

**Conclusion:**

NAAs are potent mitochondrial uncouplers in human adipocytes, independent of UCP1 and ANT. However, previously unnoticed adverse effects harm adipocyte functionality, reduce the therapeutic index of NAAs *in vitro* and therefore question their suitability as anti-obesity agents without further chemical modifications.

## Highlights

• Neutral NAAs uncouple mitochondrial respiration of intact human adipocytes.
• UCP1 and ANT are not required for NAA-uncoupling activity.
• NAAs reduce adipocyte viability, substrate oxidation capacity and ATP turnover.
• The *in vitro* therapeutic window of NAAs is smaller than that of NEN and DNP.

## Graphical abstract

**Figure:**
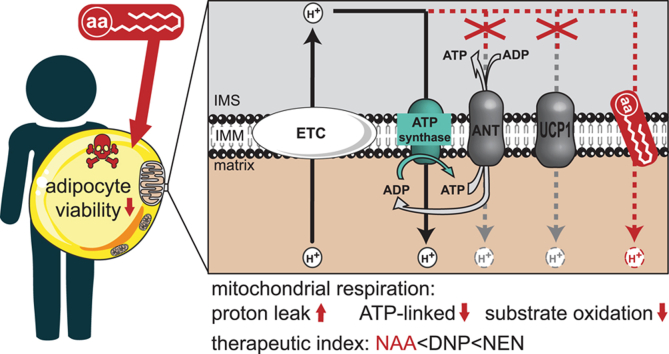

## 1 Introduction

Activation of brown adipose tissue (BAT) and induction of white adipose tissue (WAT) browning to increase energy expenditure have become attractive therapeutic targets in obesity research. The enhanced energy turnover in adipocytes can clear excessive nutrients from the circulation, thereby correcting metabolic disorders. Brown and beige adipocytes are generally considered thermogenic, energy-combusting cells, although their developmental origins are still a matter of debate [1,2]. Brown/beige adipocytes mainly use uncoupling protein 1 (UCP1), which is located in the mitochondrial inner membrane, to dissipate the proton motive force and accelerate substrate oxidation, also referred to as mitochondrial uncoupling or proton leak respiration. Therefore, UCP1 activity may improve several parameters of metabolic health in humans. For example, individuals with high BAT activity display elevated blood levels of high-density lipoprotein (HDL) cholesterol, and lower levels of glycated hemoglobin (Hb1Ac), glucose and FFA/triglycerides [[3], [4], [5]]. Studies in laboratory mice established causality and molecular mechanisms, showing that activating BAT and browning pathways of WAT in the cold improve glucose and lipid homeostasis [6,7].

In contrast to WAT mass, BAT prevalence and activity correlate negatively with age and obesity [[8], [9], [10]] and may therefore be of limited importance for the therapy of obesity and metabolic disorders in adults. Thus, discovering alternative UCP1-independent mechanisms to increase mitochondrial uncoupling has gained biomedical attention.

Chemical uncouplers of mitochondrial respiration such as niclosamide ethanolamine (NEN) and 2,4-dinitrophenol (DNP) have been recently revisited as metabolic drugs. NEN, an FDA-approved agent for tapeworm infection treatment showed promising results when repurposed to prevent and reverse high-fat diet (HFD)-induced hepatic steatosis in mice [11]. DNP, however, had been banned by the FDA due to fatal effects and narrow dose ranges [12].

Long and colleagues discovered a class of endogenous metabolites, called N-acyl amino acids (NAAs), as UCP1-independent uncouplers in mice [13]. NAAs were identified as products of the enzyme PM20D1, which catalyzes the biosynthesis and hydrolysis of NAAs from amino acids and long-chain free fatty acids [13]. The overexpression of PM20D1 in mice led to increased plasma levels of various N-oleoyl amino acids (e.g. C18:1-Phe and C18:1-Leu) and reduced diet-induced obesity, even at thermoneutrality [13]. The pharmacologic administration of C18:1-Leu reduced body mass and improved glucose tolerance in diet-induced obese mice [13]. Notably, however, these mice also had reduced food intake for unclarified reasons [13]. The uncoupling activity of NAAs was shown to be independent of UCP1 in primary murine BAT cells. In line, NAAs were able to increase oligomycin-insensitive respiration in murine C2C12 and human U2O2 cells, which are both UCP1-negative cells. As potential mitochondrial targets of NAAs, the authors found physical interaction with the adenine translocators 1 and 2 (ANT1/2), suggesting that NAAs could induce ANT's uncoupling activity [13]. In a recent report, the potency of NAAs for UCP1-independent uncoupling activity was questioned by showing less uncoupling in isolated brown fat mitochondria from UCP1–KO mice [14]. However, at least partial contribution of the ANT in NAA-mediated uncoupling was acknowledged [14]. Apparently, the NAAs are cell-permeable in mouse cells but the transport mechanisms remain elusive. Since NAAs have never been investigated in human adipocytes, the therapeutic potential of these lipidated amino acids is currently unclear. Here, we study the effects of NAAs on human adipocytes for the first time, using an established model for human subcutaneous preadipocytes and adipocytes, the SGBS cells [15,16]. Using plate-based respirometry, we assess the relation of NAAs to human UCP1 and ANT. Importantly, we reveal adverse effects of NAAs on mitochondrial function, which compromise cell viability. Relating the beneficial vs. the adverse effects of NAAs to the known chemical uncouplers DNP and NEN, we found a rather small therapeutic window for NAAs.

## 2 Material and method

### 2.1 Materials

Cell culture media and supplements were purchased from Thermo Fisher Scientific (Invitrogen, Waltham, MA, USA). All other chemicals and reagents were obtained from Sigma Aldrich (St.Louis, MO, USA), unless stated otherwise.

### 2.2 N-acyl amino acids (NAAs), 2,4-dinitrophenol (DNP) and niclosamide ethanolamine (NEN)

NAAs were synthesized as described previously [17]. DNP was purchased from Sigma, and NEN from Cayman Chemical. All compounds were dissolved in DMSO and equilibrated overnight with medium or assay buffer containing 0.4% BSA (essentially fatty acid-free) before addition to the cells. Dilutions for dose responses were made using medium/buffer containing 0.4% BSA.

### 2.3 Adipocyte culture

SGBS adipocytes were cultured and differentiated for 21 days as published [15,16]. Before all assays, cells were cultured in medium without serum and differentation cocktail for 24 h.

### 2.4 Energetic pathway studies (intact cells)

SGBS cells were differentiated in XF96-PS plates (Agilent technologies). On the day of the assay, cells were washed with XF assay medium, incubated in fresh XF assay medium containing 5 mM glucose and 2 mM glutamine (pH adjusted to 7.5) in a 37 °C air incubator for 1 h, and then subjected to respirometric analysis as previously described [16]. The following final concentrations were injected: oligomycin (1 μg/ml), the chemical uncoupler FCCP (1 μM), rotenone (4 μM), antimycin A (2 μM) and 2-deoxy-glucose (100 mM). Oxygen consumption rate (OCR) data of each well were normalized to the mean double stranded DNA content/well of all experiments (19.5 ng= ∼3000 cells). DNA was quantified using the DNAQuant-iT™ PicoGreen® dsDNA Kit (Invitrogen).

### 2.5 Energetic pathway studies (permeabilized cells)

SGBS cells were differentiated in XF96-PS plates (Agilent technologies). On the day of the assay, cells were washed with mitochondrial assay solution (MAS) and incubated in a 37 °C air incubator in fresh MAS-buffer containing 0.03% Digitonin and 0.4% fatty acid-free BSA for 15 min, followed by the respirometric analysis. The following compounds were injected (at final concentrations): 10 mM succinate, 2 μM rotenone, 4 μg/ml oligomycin, 1 mM UCP1-inhibitor GDP, 10 μM ANT-inhibitor CAT, followed by injection of the NAAs (50 μM), 1 μM FCCP and 2 μM antimycin A.

### 2.6 UCP1 mRNA and protein levels

RNA was isolated from adipocytes using the miRNeasy Mini Kit (Qiagen, Hilden, Germany, #217004). After reverse transcription (QuantiTect Reverse Transcription Kit, Qiagen,#205314), expression levels of UCP1 (fw: 5′-GCCACTCCTCCAGTCGTTA-3′, rev: 5′-TCTCTCAGGATCGGCCTCTAG-3′) and RPS13 (fw: 5′-CTTGTGCAACACCATGTGAA-3′, rev: 5′-CCCCACTTGGTTGAAGTTGA-3′) were analyzed with the CFX real-time PCR machine (Biorad) using SYBR® Green JumpStart™ Taq ReadyMix™ (Sigma, #S4438). Relative expression was analyzed using the ΔCt method.

### 2.7 Immunological detection of UCP1

Adipocytes were lysed in RIPA buffer (Sigma-Aldrich) at 4 °C for 30 min, cleared by centrifugation, and protein concentrations of the supernatant were determined using the DC Protein assay Kit (Biorad). 15 μg protein lysate were separated on a 4–12% BisTris gel (Invitrogen) and blotted onto a Nitrocellulose Membrane using iBlot (Invitrogen). The membrane was blocked in Odyssey Blocking Buffer (LiCor, Lincoln, NE USA), incubated with primary antibody against UCP1 (R&D system, #MAB6158) and beta-tubulin (#ab21057, Abcam), followed by incubation with AlexaFluor secondary antibodies (#ab175774 and #ab175784, Abcam, Cambridge, England). Signals were detected using the Odyssey FC (LiCor).

### 2.8 Viability

SGBS cells were seeded and differentiated in 96-well-plates. Cells were incubated with NAA concentrations as indicated in the figures. After 21 h, cell viability was determined using the 3- [4,5] dimethylthiazol-2,5-diphenyl tetrazolium bromide (MTT) assay: 1 mg/ml MTT in phenol red-free medium was added to the well, and the cells were incubated at 37 °C for 3 h. An equal volume of isopropanol was added per well to dissolve formazan, followed by absorbance reading at 550 nm in a plate reader (EnSpire). The cell-free wells served as background values. Data are the mean of 3–5 independent experiments, each performed in 4–8 wells/condition. Percent viability was calculated using vehicle (DMSO) as control: % viability = (100 x (sample/control)).

### 2.9 Statistics

**Fig. 1:**
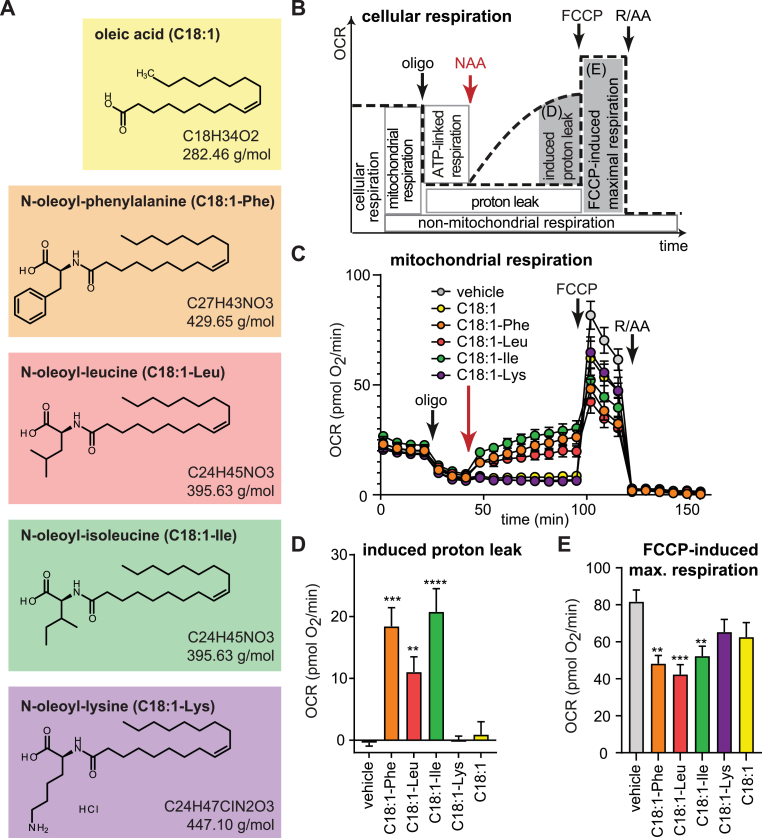
NAAs with neutral amino acid uncouple intact human adipocytes respiration Respiration measurements of intact human subcutaneous (SGBS) adipocytes to address uncoupling effects of N-acyl amino acids (NAAs). (A) The following compounds were used: oleoyl-l-phenylalanine (C18:1-Phe, 50 μM), oleoyl-l-leucine (C18:1-Leu, 50 μM), oleoyl-l-Isoleucine (C18:1-Ile, 50 μM) oleoyl-l-lysine (C18:1-Lys, 50 μM) and oleic acid (C18:1, 50 μM). Data for free amino acids (all 50 μM) are presented in Fig. S1 (B) Scheme of the oxygen consumption rates (OCR) time trace, depicting partitioning of different respiratory modules. (C) Mitochondrial OCR traces after subtraction of non-mitochondrial OCR. (D) Induced proton leak respiration after compound injection. (E) Maximal substrate oxidation (respiration) after FCCP (1 μM) injection. Non-mitochondrial OCR after rotenone and antimycin A injection are shown in Fig. S2. OCR of each well are normalized to mean DNA content/well of all plates (19.5 ng/well) and represent means ± SEM of 4–6 independent plates. Statistical differences were determined using one-way ANOVA (posthoc: Dunnett's) and are indicated: *p < 0.05, **p < 0.01, ***p < 0.001, ****p < 0.0001 vs vehicle.

**Fig. 2:**
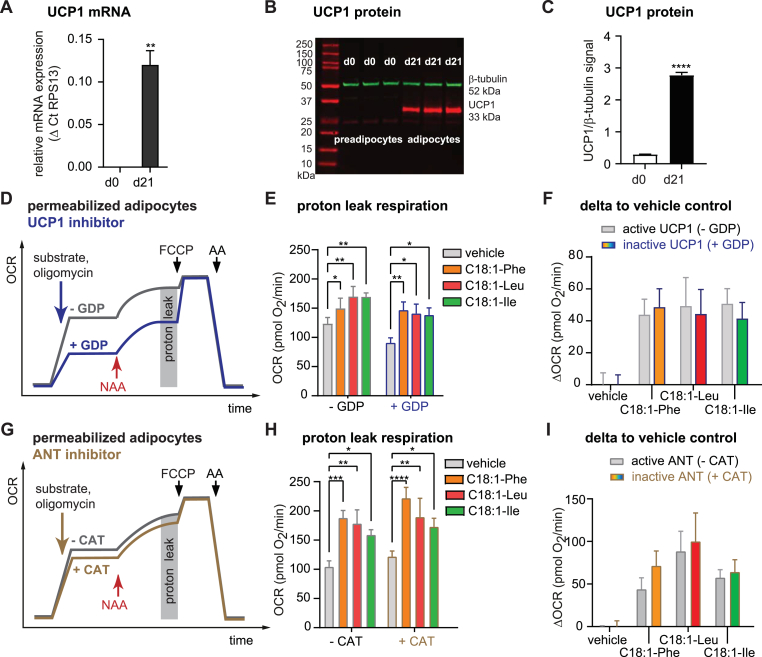
NAAs uncouples respiration independent of UCP1 and ANT (A) UCP1 mRNA and (B,C) protein level in SGBS preadipocytes (d0) and adipocytes differentiated for 21 (d21) days. Data represent the mean ± SEM of 3 independent differentiations. (D) Scheme of OCR time trace depicting the experimental approach to determine the contribution of UCP1 to NAA-induced proton leak respiration using the UCP1-inhibitor GDP (1 mM) and succinate (10 mM) as the substrate. Mitochondrial OCR traces of the permeabilized adipocytes (subtracted non-mitochondrial OCR, Fig. S4A) are shown in Fig. S4D). (E) Proton leak respiration in adipocytes after addition of NAAs (50 μM, grey box in D) in the presence or absence of GDP. (F) Proton leak OCR are presented as delta to the mean of the vehicle control for each individual plate. No significant difference was detected between -GDP vs + GDP for each compound. (E,F) Data are the mean ± SEM of 12–29 wells measured on 8 different plates of 5 independent differentiations. Statistical differences were determined using two-way ANOVA (posthoc: Dunnett's, Bonferroni). *p < 0.05, **p < 0.01, ***p < 0.001, ****p < 0.0001 vs vehicle. (G) Scheme of the OCR time trace depicting the experimental approach to determine the contribution of ANT to NAA-induced proton leak respiration by blocking its uncoupling activity with CAT (10 μM), using succinate (10 mM) as the substrate. Mitochondrial OCR traces of the permeabilized adipocytes (subtracted non-mitochondrial OCR; Fig. S4F) are shown in Fig. S4I. (H) Proton leak respiration in adipocytes after the addition of NAAs (50 μM, grey box in G) in the presence or absence of CAT. (I) Proton leak OCR are presented as delta to the mean of the vehicle control for each individual plate. No significant difference was detected between -CAT vs + CAT for each compound. (H, I) Data are the mean ± SEM of 10–30 wells measured on 8 different plates of 5 independent differentiations. Statistical differences were determined using two-way ANOVA (posthoc: Dunnett's, Bonferroni). *p < 0.05, **p < 0.01, ***p < 0.001, ****p < 0.0001 vs vehicle.

**Fig. 3:**
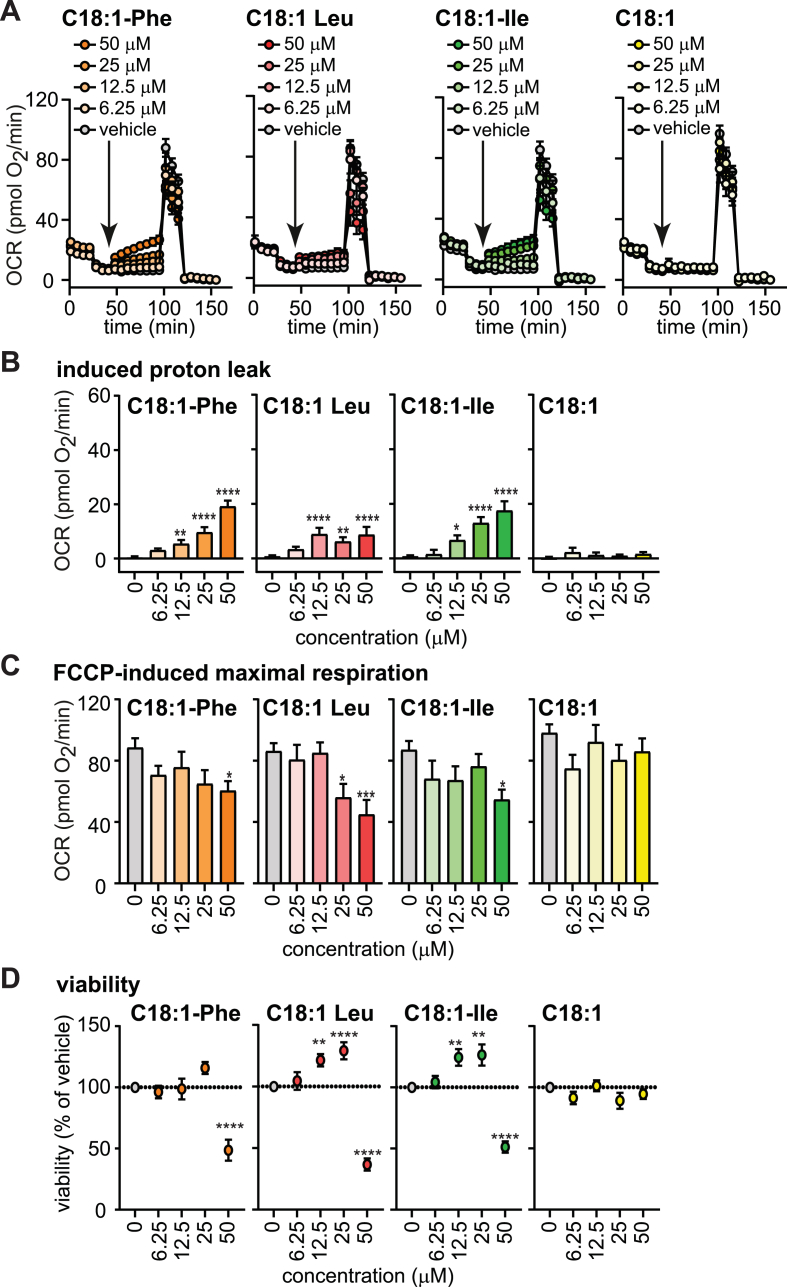
Dose-responses of proton leak, maximal respiration and adipocyte viability to NAAs (A-C) Respiration measurements to address respiratory dose-responses to NAA in intact human adipocytes. (A) Mitochondrial OCR traces of the adipocytes (B) Induced proton leak respiration. (C) FCCP-induced maximal respiration. All OCR data are normalized to mean DNA content/well of all plates (19.5 ng/well) and represent means ± SEM of 10–44 wells measured on 4 independent plates Statistical differences were determined using one-way ANOVA (posthoc: Dunnett's) and are indicated: *p < 0.05, **p < 0.01, ***p < 0.001, ****p < 0.0001 vs vehicle. (D) Viability of adipocytes after exposure to NAAs for 21 h was assessed as described in Methods. Light-microscopy of adipocytes after 21 h with compound incubation are shown in Fig. S5C. Data are the mean ± SEM of 24–57 wells measured on 3–5 independent adipocyte differentiations and are presented as % of vehicle (DMSO) control. Statistical differences were determined using one-way ANOVA (posthoc: Dunnett's) and are indicated: *p < 0.05, **p < 0.01, ***p < 0.001, ****p < 0.0001 vs vehicle.

**Fig. 4:**
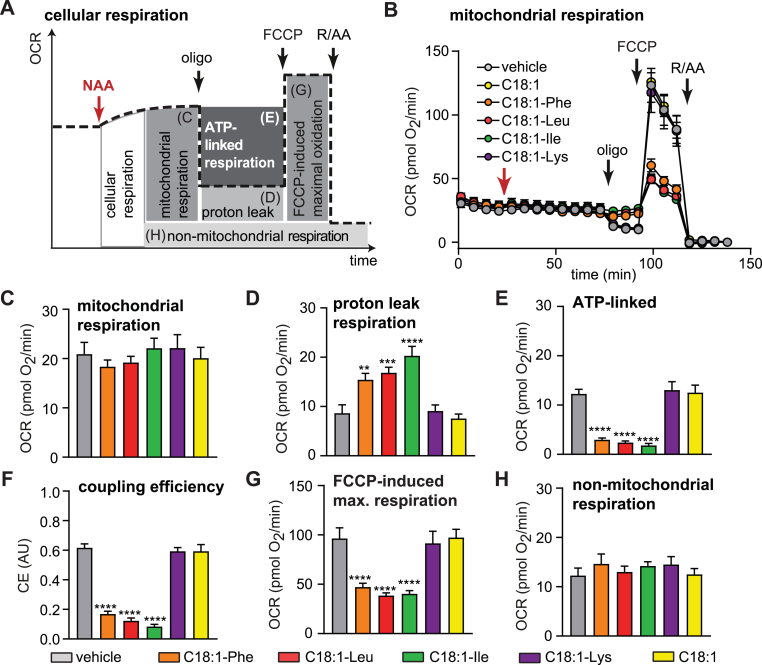
NAAs with neutral amino acid compromise ATP-linked respiration in human adipocytes (A) Scheme of the OCR time trace depicting the experimental approach to determine the effects of NAAs on basal and ATP-linked respiration in intact human adipocytes. (B) Mitochondrial OCR traces after subtraction of non-mitochondrial OCR. (C) Mitochondrial respiration after compound addition (mean of two time points before oligomycin injection). (D) Proton leak respiration. (E) ATP-linked respiration. (F) Coupling efficiency, defined as fraction of mitochondrial OCR dedicated to ATP synthesis. (G) FCCP-induced maximal respiration. (H) Non-mitochondrial OCR after addition of rotenone and antimycin A. All OCR data are normalized to mean DNA content/well of all plates (19.5 ng/well) and are presented as means ± SEM of 11–16 independent wells measured of three independent adipocyte differentiations. Statistical differences were determined using one-way ANOVA (posthoc: Dunnett's) and are indicated: *p < 0.05, **p < 0.01, ***p < 0.001, ****p < 0.0001 vs vehicle.

**Fig. 5:**
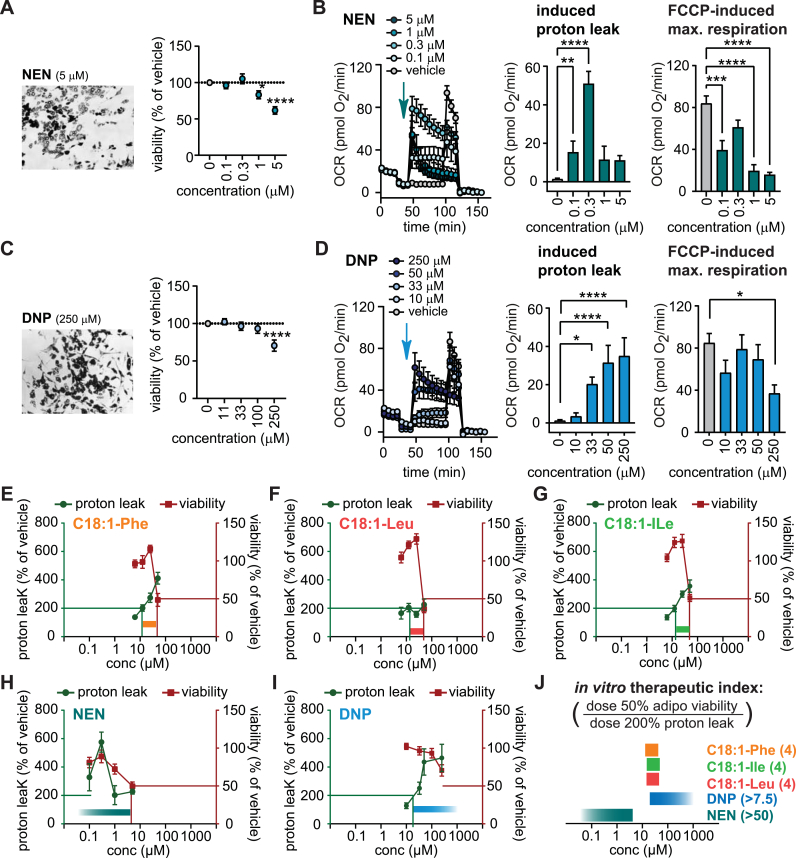
NAA display a smaller *in vitro* therapeutic window than NEN and DNP Light microscopy and viability of the adipocytes after exposure to indicated concentrations of (A) NEN and (C) DNP for 21 h. Data are the mean ± SEM of 32–57 wells measured of 3–5 independent differentiations Statistical differences were determined using one-way ANOVA (posthoc: Dunnett's) and are indicated as * p < 0.05, **p < 0.01, ***p < 0.001, ****p < 0.0001 vs vehicle. (B) NEN and (D) DNP effects on adipocyte respiration, extracting induced proton leak and effects on FCCP-induced maximal respiration. All OCR data are normalized to mean DNA content/well of all plates (19.5 ng/well) and represent means ± SEM of 5–25 wells measured on 1–3 independent plates. Statistical differences were determined using one-way ANOVA (posthoc: Dunnett's) and are indicated: *p < 0.05, **p < 0.01, ***p < 0.001, ****p < 0.0001 vs vehicle. (E–J) Therapeutic window for NAAs. (E-G, data are from the main Fig. 3), NEN (H, data are from A, B) and DNP (I, data are from C, D). (J) We estimated the *in vitro* therapeutic index as the ratio of the dose resulting in 50% reduction in adipocyte viability and the dose resulting in a 2-fold increase (200%) of proton leak.

To test for group differences shown in Fig. 2A and C, the unpaired *t*-test (two-tailed) was used. One-way ANOVA (post-hoc: Dunnett) was performed for Fig. 1D–E, 3B-D, 4C–H and 5A-D. For Fig. 2E, F, H and I, two-way ANOVA was performed (post-hoc: Dunnett, Bonferroni). p < 0.05 was considered statistically significant. All statistical tests were performed using Graph Pad Prism (Version 10).

## 3 Results

### 3.1 N-acyl amino acids induce proton leak respiration in intact human adipocytes

To determine the uncoupling activity of NAAs on human adipocytes, four different NAA derivatives were synthesized (Fig. 1A). The identical unsaturated fatty acid moiety oleate (C18:1) was used, based on high uncoupling bioactivity [18], and the amino acid residues were either neutral (Phe, Ile and Leu), previously shown as potent uncouplers in mice, or charged (Lys), previously shown not to uncouple respiration [18]. We determined the bioenergetic effects of these four NAAs in SGBS cells, an established human adipocyte model system with a well-characterized bioenergetic profile similar to primary subcutaneous adipocytes [15,16,19]. SGBS adipocytes, differentiated for 21 days, were assessed by plate-based respirometry. 50 μM of NAA, oleate or vehicle control were acutely injected after ATP-synthase inhibitor oligomycin injection to obtain NAA-induced proton leak respiration (Fig. 1B–C). NAAs with neutral amino acids, C18:1-Phe C18:1-Leu and C18:1-Ile, showed significant induction of proton leak by 351%, 256% and 253%, respectively (Fig. 1D). In contrast, the charged C18:1-Lys, free oleic acid (C18:1), vehicle control (Fig. 1D), as well as free amino acids (Fig. S1) did not induce proton leak respiration. Notably, C18:1-Ile, C18:1-Phe and C18:1-Leu reduced FCCP-induced maximal respiration (Fig. 1E), but did not impact non-mitochondrial respiration (Fig. S2).

### 3.2 NAAs induce proton leak independent of human UCP1

The involvement of UCP1 in NAA-induced proton leak remains controversial. Two studies show NAA-induced proton leak respiration in UCP1-negative cells, such as brown and beige adipocytes from UCP1-knockout mice [13,17], whereas others found reduced NAA-dependent respiration in isolated brown fat mitochondria of UCP1-knockout mice [14]. Here, we demonstrate NAA-induced proton leak respiration in UCP1-negative human cells (i.e.: SGBS preadipocytes and human macrophage-like THP1 cells, Fig. S3) and UCP1-positive SGBS adipocytes. At day 21 of differentiation, SGBS adipocytes show high UCP1 mRNA (Fig. 2A) and protein levels (Fig. 2B and C). The differentiated adipocytes were permeabilized to enable UCP1 inhibition using GDP (Fig. 2D). Before adding NAAs, proton leak respiration partially depends on UCP1 activity (∼32%), seen as lower respiration rates in the presence of GDP (Fig. S4B). The addition of NAAs is indicated by the red arrow (Fig. 2D, S4D). The neutral NAAs C18:1-Phe, C18:1-Leu, and C18:1-Ile significantly increase proton leak respiration in the presence of GDP (Fig. 2E and F), indicating that the induction of uncoupling activity is independent of UCP1.

### 3.3 NAAs induce proton leak independent of ANT uncoupling activity

Previous data on the physical interaction between NAAs and the ANT suggested a link between NAA-induced uncoupling and ANT's protonophoric function [13]. Fatty acid-induced protonophoric activity of the ANT is a property seen in isolated mitochondria, and can be prevented by the highly selective and potent ANT-inhibitor carboxyatractyloside (CAT) or adenine nucleoside di- and triphosphates [20], the latter suggesting minor physiological relevance in intact cells given millimolar concentrations of ATP. Others show partial reduction of NAA-induced proton leak by CAT in isolated BAT and liver mitochondria, suggesting effects similar to fatty acid-induced ANT uncoupling [14]. To test if the ANT is important for NAA-uncoupling in human adipocytes, the cells were carefully permeabilized to enable access of CAT to mitochondria to lock the ANT in the non-conductive c-state [21] (Fig. 2G). Permeabilization did not disrupt the mitochondrial outer membrane (as judged by cytochrome C injection, red symbol Fig. S4E) and CAT fully blocked ADP/ATP exchange during state 3 respiration (brown symbol, Fig. S4E). Blocking the human ANT with CAT had no effect on proton leak respiration in adipocytes (Fig. S4G). The NAAs were injected in the presence or absence of CAT (Fig. 2G, Fig. S4I, indicated by the red arrow). Irrespective of CAT, C18:1-Phe, C18:1-Leu, and C18:1-Ile induced proton leak respiration (Fig. 2H and I). Thus, the ANT is not essential for NAA-induced uncoupling in human adipocytes.

### 3.4 Adverse effects of NAAs on substrate oxidation

We found that 50 μM of neutral NAAs suppress FCCP-induced maximal respiration in human adipocytes (Fig. 1C,E), consistent with noted observations in mouse beige and brown adipocytes [17]. Next, we comprehensively characterized the NAA dose-responses of beneficial (uncoupling) vs. adverse effects (suppression of substrate oxidation) (Fig. 3A). While C18:1-Phe, C18:1-Leu, and C18:1-Ile induced uncoupling activity above 6.25 μM (Fig. 3B), significant reductions of FCCP-induced maximal respiration were seen at 25–50 μM (Fig. 3C). Next, the MTT assay was used to assess cell viability of preadipocytes (Figs. S5A and B) and adipocytes (Fig. 3D, Fig. S5C), which were exposed to various concentrations of NAAs for 21 h. As judged by light microscopy, incubation with 50 μM of C18:1-Phe, C18:1-Leu, and C18:1-Ile resulted in a robust loss of adherent adipocytes (Fig. S5C) and the complete loss of metabolically active preadipocytes (Figs. S5A and B). Thus, beneficial uncoupling effects of NAAs were outweighed by the adverse effects on viability and mitochondrial oxidation at concentrations above 25 μM (Fig. 3C,D).

### 3.5 NAAs decrease ATP-linked respiration in adipocytes

Although 50 μM NAA suppress mitochondrial oxidation, the reduced maximal oxidation rates did not compromise basal cellular respiration, leaving unused spare capacity for adipocyte function. Therefore, to further understand the adverse effects on cell viability, the adipocytes were treated with the respective compounds before the administration of oligomycin, enabling us to determine the effects of NAAs on ATP turnover (Fig. 4A and B). The NAAs did not acutely increase basal respiration rates (Fig. 4C), but increased oligomycin-insensitive proton leak (Fig. 4D). This resulted in significantly reduced ATP-linked respiration (Fig. 4E). The addition of NAAs was accompanied by a slight increase in glycolytic acidification (Fig. S6), suggesting at least the attempt to maintain ATP homeostasis. Coupling efficiency (CE) of the adipocytes, representing the fraction of mitochondrial respiration that is coupled to ATP production, showed robust decreases for neutral NAAs, from mean values of 0.63 (vehicle), 0.59 (C18:1-Lys) and 0.58 (oleic acid) to mean values of 0.17 (C18:1-Phe), 0.14 (C18:1-Leu), 0.09 (C18:1-Ile) (Fig. 4F). The changes in the experimental setup, swopping oligomycin and NAA-injection, did not alter the previously seen effects on FCCP-induced maximal respiration (Fig. 4G, c f. Fig. 1E) and non-mitochondrial respiration (Fig. 4H, c f. Fig. S2). The results demonstrate that neutral NAAs significantly reduce mitochondrial ATP production, possibly causing a bioenergetic crisis that may lead to decreased viability already after 21 h.

### 3.6 NAAs display a smaller therapeutic index than DNP and NEN

To better judge the therapeutic value of the novel uncoupler NAA, we compared our compounds to niclosamide ethanolamine (NEN) and 2,4-dinitrophenol (DNP), two established uncoupling agents. Viability assays and bioenergetic profiles were performed for various doses of NEN (Fig. 5A–B) and DNP (Fig. 5C–D). 5 μM of NEN reduced viability by 40% (Fig. 5A), paralleled by 80% reduced FCCP-induced maximal respiration (Fig. 5B) but 0.3 μM of NEN maximized proton leak with minor effects on viability and oxidation (Fig. 5B). Regarding DNP, only a very high dose of 250 μM reduced viability by 30% (Fig. 5C), paralleled by 56% reduced FCCP-induced maximal respiration (Fig. 5D). We calculated the *in vitro* therapeutic index using a 2-fold increase (200%) of proton leak as beneficial threshold, and a 50% reduction in viability as adverse threshold (Fig. 5E–J), depicting a rather narrow therapeutic window for NAAs as compared to NEN and DNP.

## 4 Discussion

NAAs as uncouplers of mitochondrial respiration in human adipose tissue are attractive candidates for the treatment of obesity and other metabolic disorders. Among the unknowns, such as the mechanism of cellular uptake, molecular mechanism and dose-responses, NAAs have never been tested in human adipocytes. We show that NAA are taken up by intact human adipocytes and potently uncouple mitochondria in a dose-dependent manner (Fig. 1, Fig. 3). Consistent with previous mouse data, equimolar concentrations of oleate fail to induce uncoupled respiration [13]. Similarly, equimolar concentrations of the respective free amino acids do not uncouple mitochondria. In contrast to reports observing no functional differences between NAA and free fatty acids [14], we conjugated our compounds to albumin for best practice to avoid precipitation of the lipophilic compounds in the medium and uncontrolled penetration of the plasma membrane.

NAAs’ uncoupling action does neither require UCP1 nor the uncoupling property of the ANT (Fig. 2). The previously observed partial involvement of ANT-mediated uncoupling in isolated mouse mitochondria [14] is absent in permeabilized human adipocytes. Notably, however, the uncoupling activity through the ANT has so far mainly been observed in isolated mitochondria, which are completely stripped off purine nucleotides such as ADP and ATP, which act also as inhibitors of the uncoupling property [20]. Residual ADP and ATP traces in our carefully permeabilized human adipocytes may have prevented ANT-uncoupling. This, however, represents the physiologically more relevant state in the eukaryotic cell. The physical interaction of NAA and ANT may still impact ANT function, but our data demonstrate that this is not essential for the uncoupling function.

Since other chemical uncouplers such as DNP can exhibit toxic effects, usually explained by a narrow window of the therapeutic dose [22], we explored the adverse effects of NAA. Notably, active NAAs showed sharp threshold concentrations for reduced viability of preadipocytes and adipocytes, suggesting an even narrower therapeutic dose window than DNP and NEN. The reduced adipocyte viability was paralleled by declines in FCCP-induced maximal respiration. Since FCCP-induced maximal respiration is decreased by high concentrations of NAA, these compounds could interfere either with the substrate delivery upstream of mitochondrial oxidation, or with mitochondrial electron transport chain complexes.

Furthermore, we find that NAAs compromise ATP-linked respiration and mitochondrial coupling efficiency (Fig. 4) which cannot be fully explained with the reduction in oxidation, as oxidation capacity was still sufficient for basal adipocyte respiration. Notably, ATP-linked respiration is controlled by decreased cellular ATP consumption, impaired ADP/ATP exchange or ATP synthase activity. Thus, previous findings on the interaction of NAA with the ANT [13] could impact ADP/ATP exchange and thus, cellular function and viability. In mouse cells, ATP-linked respiration appears to be less sensitive towards NAAs and the additional NAA-mediated uncoupling increases resting OCRs [17], which would be a prerequisite to induce effects on weight loss and/or glucose-lipid homeostasis. The *in vitro* therapeutic index comparing NAAs, DNP and NEN suggests a narrower dose window for NAAs, warranting caution for translational efforts.

### 4.1 Conclusion

Altogether, our results indicate effective uncoupling activity of NAAs in human adipocytes but the narrow therapeutic dose range may require chemical modifications to advance these novel endogenous uncouplers to the clinic.

## Declaration of competing interest

None.

## Data Availability

Data will be made available on request.
