# Supplementary material for: Adverse bioenergetic effects of N-acyl amino acids in human adipocytes overshadow beneficial mitochondrial uncoupling
Source: Redox Biol. 2023 Sep 2;66:102874. doi: 10.1016/j.redox.2023.102874 (PMC10493596; doi:10.1016/j.redox.2023.102874)
Supplement: Multimedia component 1 [file mmc1.pdf]

Appendix A. Supplementary data

Adverse bioenergetic effects of N-acyl amino acids in human adipocytes overshadow beneficial mitochondrial uncoupling

Marie Herrnhold[1], Isabel Hamp[2], Oliver Plettenburg[2], Martin Jastroch[1], Michaela Keuper[1#]

[1] Department of Molecular Biosciences, The Wenner-Gren Institute, The Arrhenius Laboratories F3, Stockholm University, SE-106 91 Stockholm, Sweden

[2] Institute of Medicinal Chemistry, Helmholtz Zentrum München, German Research Center for Environmental Health (GmbH), Neuherberg, Germany; Institute of Organic Chemistry, Leibniz Universität Hannover, Hannover, Germany

[#]correspondence to:

Michaela Keuper, michaela.keuper@su.se

Running title: NAA effects on human adipocytes

Content:

Figure S1-S6

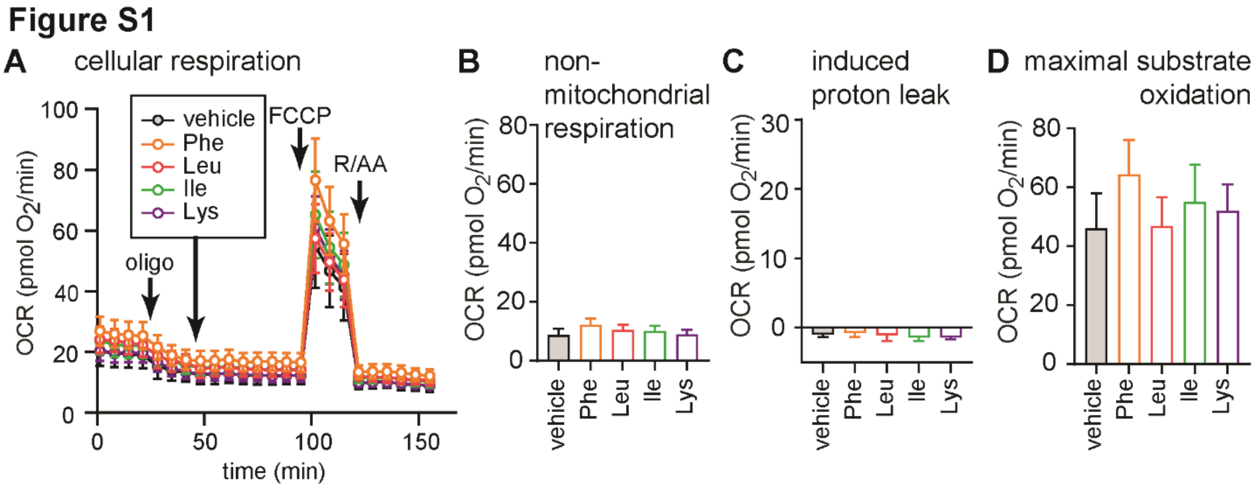

Figure S1: Free amino acids do not change the bioenergetics of human subcutaneous SGBS adipocytes

(A) Cellular OCR traces of the adipocytes. After blocking ATP-synthase with oligomycin, compounds were added to obtain the compound-induced proton leak respiration. The following compounds and concentrations were used: vehicle (H2O), phenylalanine (Phe, 50 µM), leucine (Leu, 50 µM), Isoleucine (ILe, 50 µM) and lysine (Lys, 50 µM). **(B)** Non-mitochondrial OCR were obtained after addition of rotenone (R, 4 µM) and antimycin A (AA, 2 µM), and were subtracted from all OCR (cellular respiration) to obtain mitochondrial respiration. **(C)** Compound-induced proton leak respiration. **(D)** Maximal substrate oxidation. OCR data for each well are normalized to 19.5 ng dsDNA and are the mean + SEM of 7-8 independent stimulated wells measured on one plate. No statistical differences were detected using one-way ANOVA (posthoc: Dunnett’s).

Figure S2

A

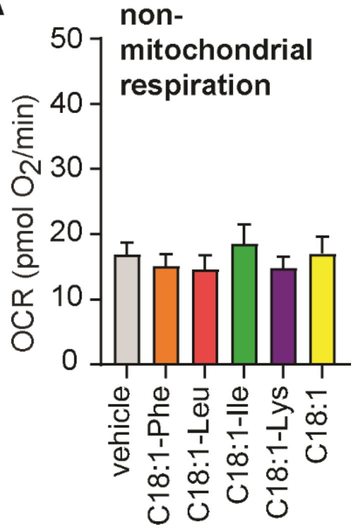

Figure S2: NAAs has no effect on non-mitochondrial respiration

Non-mitochondrial OCR were obtained by injecting Rotenone (R, 4 µM) and Antimycin A (AA, 2 µM) at the end of the respirometry and were subtracted from all other OCR presented in main Figure 1.

Figure S3

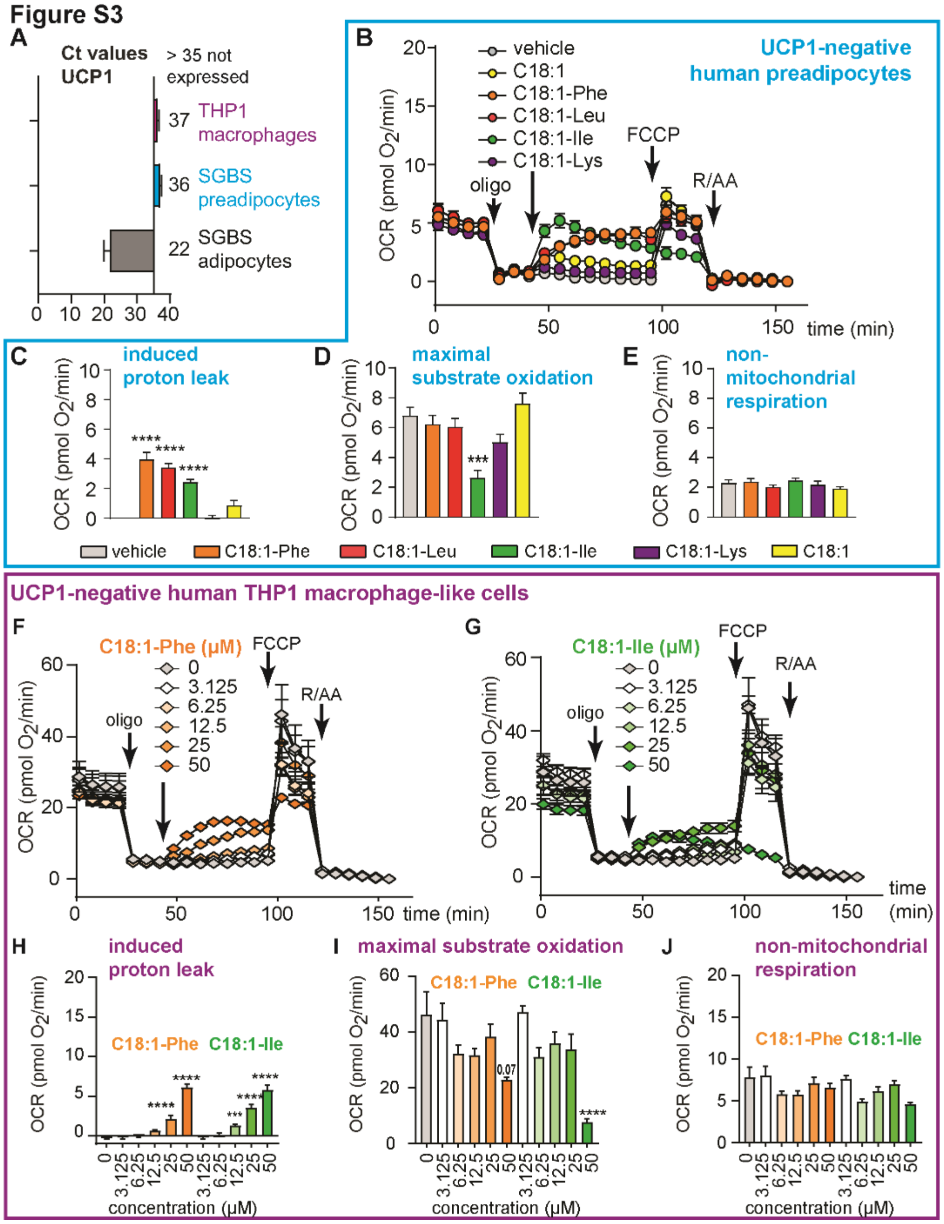

Figure S3: NAAs with neutral amino acid residue uncouple UCP1-negative cells (intact human SGBS preadipocytes and human macrophage-like THP1)

(A) qPCR confirming no expression of UCP1 in SGBS preadipocytes and THP1 macrophages. Respiration measurements to address uncoupling effects of N-acyl amino acids (NAAA) in intact human subcutaneous SGBS preadipocytes **(B-E)** and human THP1 macrophages **(F-J)** were performed as depicted in Figure 1A. (**B,F,G**) OCR time traces after subtracting non-mitochondrial OCR **(E,J)** which were obtained after addition of Rotenone (R, 4 µM) and Antimycin A (AA, 2 µM). After inhibiting ATP-synthase with oligomycin (1 µg/ml), compounds were added to obtain the induced proton leak respiration. (**C, H)** The addition of the chemical uncoupler FCCP (1 µM) allow for estimations of maximal substrate oxidation **(D, I).** OCR data for each well are normalized to same DNA content (19.5 ng) as d21 adipocytes (see Figure 1) and are presented as means ± SEM of 8-14 (**B-E**) or 3-8 (**F-J**) independent stimulated wells, each on one plate. Statistical differences were determined using one-way ANOVA (posthoc: Dunnett's) and are indicated: * $p < 0.05$, ** $p<0.01$, *** $p< 0.001$, ****$p<0.0001$ vs vehicle.

Figure S4

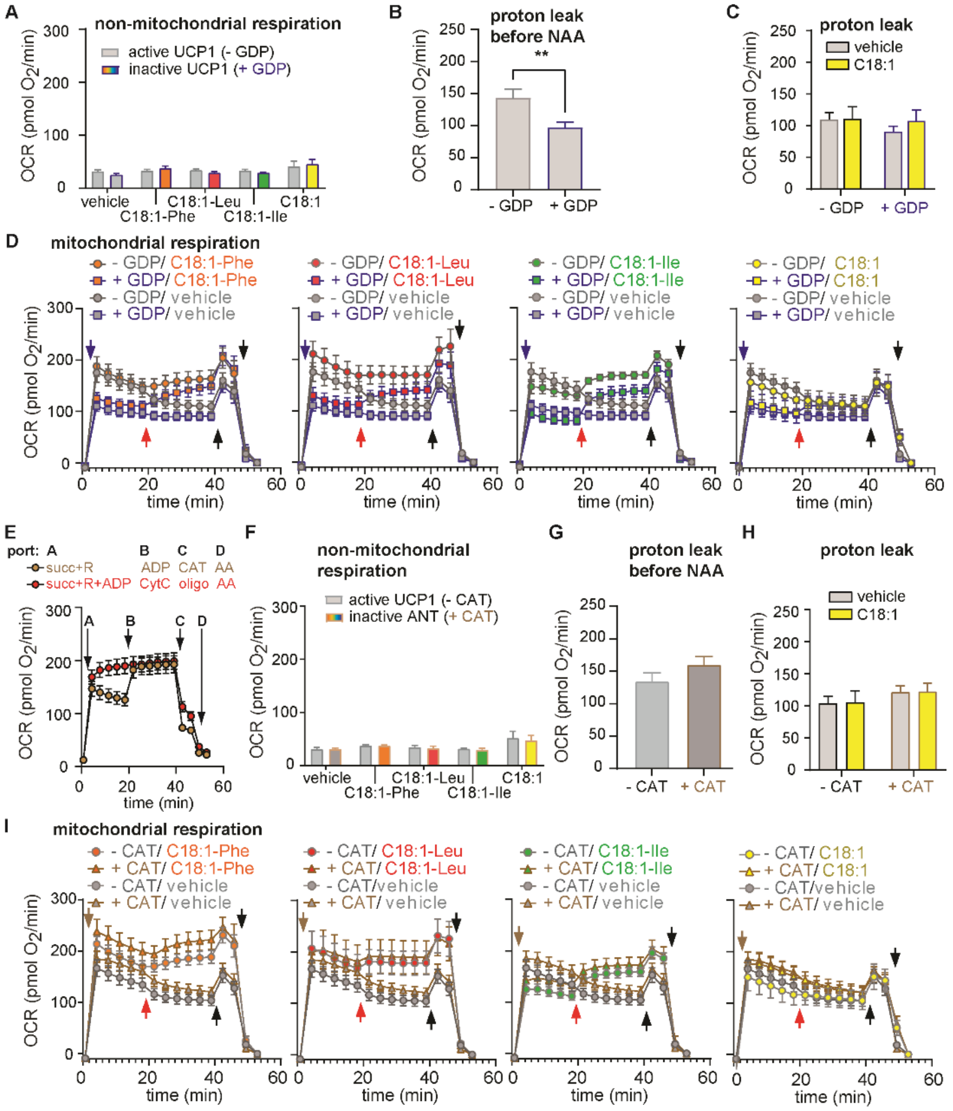

Figure S4: No effect of GDP and CAT on non-mitochondrial respiration in permeabilized SGBS adipocytes

(A) Non-mitochondrial OCR were obtained by injecting Antimycin A (AA, 2 µM) at the end of the respirometry and were subtracted from all other OCR presented in main Figure 2. **(B)** Proton leak respiration before addition of NAA (GDP effect only). **(C)** Proton leak respiration after addition of vehicle and oleic acid (C18:1) indicated as red arrow in the OCR time traces **(D). (A-D)** Data are the mean of 12-29 wells measured on 8 different plates of 5 independent adipocyte differentiations. **(E)** Control experiment showing that 10 µM CAT inhibits ANT-respiration (brown symbol) and that the outer mitochondrial membrane is still intact (red symbols).The final concentrations were: succinate (10 mM, succ), rotenone (2 µM, R), ADP (4 mM), CAT (10 µM), Cytochrome C (10 µM, CytC), oligomycin (4 µg/ml, oligo) and Antimycin A (2 µM, AA). **(F)** Non-mitochondrial OCR were obtained by injecting AA at the end of the respirometry and were subtracted from all other OCR presented in main Figure 2. **(G)** Proton leak respiration before addition of NAA (CAT effect only). **(H)** Proton leak respiration after addition of vehicle and oleic acid (C18:1) indicated as red arrow in the OCR time traces **(I). (F-I)** Data are the mean of 10-30 wells measured on 8 different plates of 5 independent adipocyte differentiations.

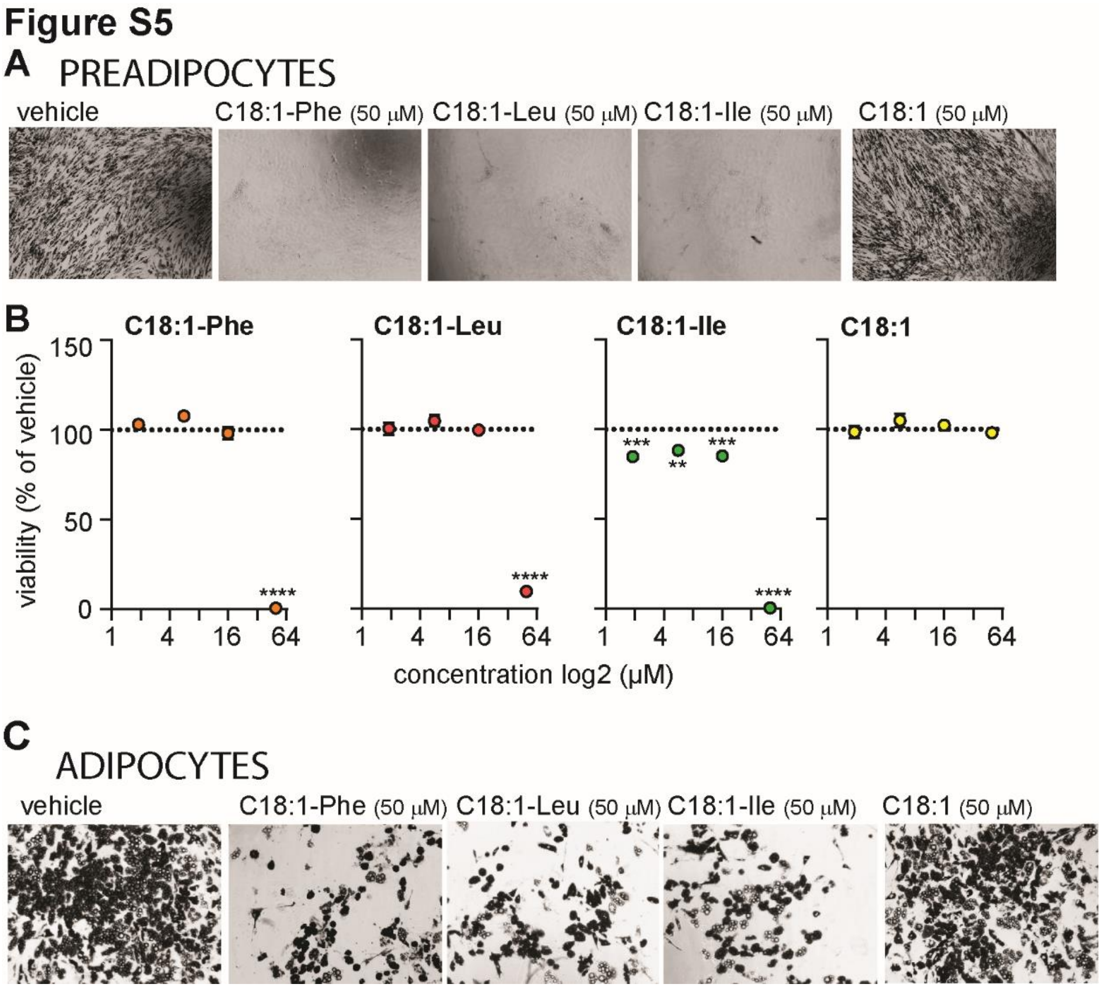

Figure S5: NAA reduce viability of preadipocytes.

(A,B) Preadipocytes were exposed to the indicated compounds for 21h followed by MTT assay. **(A)** Microscopic pictures showing loss of adherent cells and complete loss of metabolic active preadipocytes with 50 µM of NAA that induce proton leak respiration. **(B)** MTT was measured at OD 550 nm and is presented as % of vehicle (DMSO) control. Data are the mean ± SEM of 24 wells measured on 3 independent days. Statistical differences were determined using one-way ANOVA (posthoc: Dunnett's) and are indicated: * $p < 0.05$, ** $p<0.01$, *** $p< 0.001$, ****$p<0.0001$ vs vehicle. **(C)** Microscopic pictures were taken from adipocytes that had been exposed to the indicated compounds for 21h followed by MTT assay. MTT was measured at OD 550 nm and is presented in main Figure 3D.

Figure S6

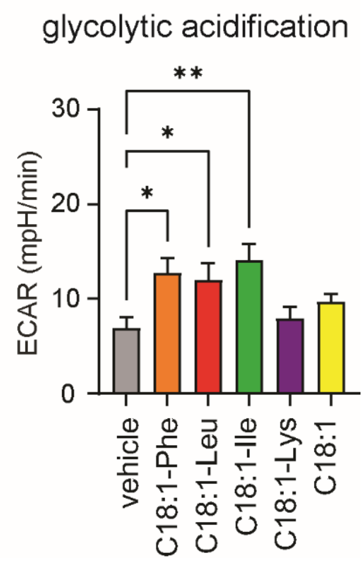

Figure S6: NAAs with neutral amino acid induce a compensatory increase in glycolytic acidification in human adipocytes

Glycolytic ECAR (mean of last two values before oligomycin injection) after subtracting the non-glycolytic ECAR (lowest value after addition of 2-deoxy-glucose). Data are presented as means ± SEM of 11-16 independent wells measured of three independent adipocyte differentiations. Statistical differences were determined using one-way ANOVA (posthoc: Dunnett's) and are indicated: * $p < 0.05$, ** $p<0.01$, *** $p< 0.001$, ****$p<0.0001$ vs vehicle.
